# Supplementary material for: Low-Grade Adenosquamous Carcinoma of the Breast: A Single-Center Retrospective Study and a Systematic Literature Review
Source: Cancers (Basel). 2024 Dec 20;16(24):4246. doi: 10.3390/cancers16244246 (PMC11674631; doi:10.3390/cancers16244246)
Supplement: Supplementary file 1 [file cancers-16-04246-s001.zip › Supplementary File S1.pdf]

Supplementary File S1

The histopathological images and pathological description from Patients 2 and 3. The histopathological samples from Patient 1 have not been preserved, but description by the pathologist has been provided.

Patient 1

In the sections, a 20 mm lesion corresponding to the macroscopic change is observed in all slices. In the central part of the lesion, within the connective tissue, tubular structures that are slightly dilated and branching are present, surrounded by a small amount of loose connective tissue. At the peripheral parts of the lesion, the tubular structures appear narrow and infiltrative in growth pattern, with some cellular strands without lumen formation. In some tubules or strands, slight differentiation towards squamous epithelium is seen. Mild to moderate nuclear atypia is present, along with a few mitoses. Around the strands, there is newly formed connective tissue, which in places is accompanied by moderate lymphocytic infiltration. Between the strands, normally structured lobular or ductal structures remain.

Additionally, in two slices at the 7–8 o'clock position adjacent to the lesion, an 8 mm area contains abnormal, dilated ducts lined by cribriform ductal-type epithelium, where nuclear atypia ranges from moderate to severe.

In the immunohistochemical staining performed on the lesion area, the tubular structures and cellular strands are predominantly CK5/6-positive. Around the tubules or strands, there is typically a calponin- and p63-positive myoepithelial cell layer; however, in some areas, the myoepithelium is discontinuous, and in a few strands, the staining result is negative, with the myoepithelium absent. In the newly formed stroma, calponin-positive spindle-shaped reactive cells are observed. In CK7 staining, the epithelial cells on the luminal side of the tubular structures stain strongly positive, while the surrounding myoepithelial-like cells stain only weakly. No CK7-positive cells are seen in the stroma. The cells are CD117-negative. Estrogen receptor staining is largely negative, with up to 50% of cells showing positivity in some areas. Progesterone receptor staining is negative, as is Her2 staining (0). The proportion of proliferative cells is 10%.

Patient 2

The samples taken from the tumor show infiltrative tumor tissue with maximum dimensions of 30 x 26 mm. The stroma of the tumor tissue varies in cellularity; at the peripheral areas, cellularity is low with fibrotic connective tissue, whereas in the central areas, cellularity is quite high with nuclear atypia, occasional pleomorphic multinucleated cells, and a moderate number of mitoses. Within the stroma, epithelial strands are observed, showing squamous differentiation, and at the periphery, individual tubular structures are also present. Nuclear atypia in the epithelial cells is predominantly moderate.

In the surrounding tissue, benign intraductal papillomatosis is seen, with papillomas varying in size from 1–4 mm. Additionally, fibrocystic changes are present, including some columnar cell changes, ductal calcifications, and small foci of sclerosing adenosis. In CK-PAN staining, the morphologically epithelial nests are strongly positive, while the spindle-like cells also show cytokeratin positivity in some areas, as well as SMA positivity. SMA-positive cells are also observed surrounding the epithelial nests.

The cells are largely hormone receptor-negative, though focally 10% of the cells are positive for both estrogen and progesterone receptors. Her2 staining is negative (0). The proportion of proliferative cells is 20%.

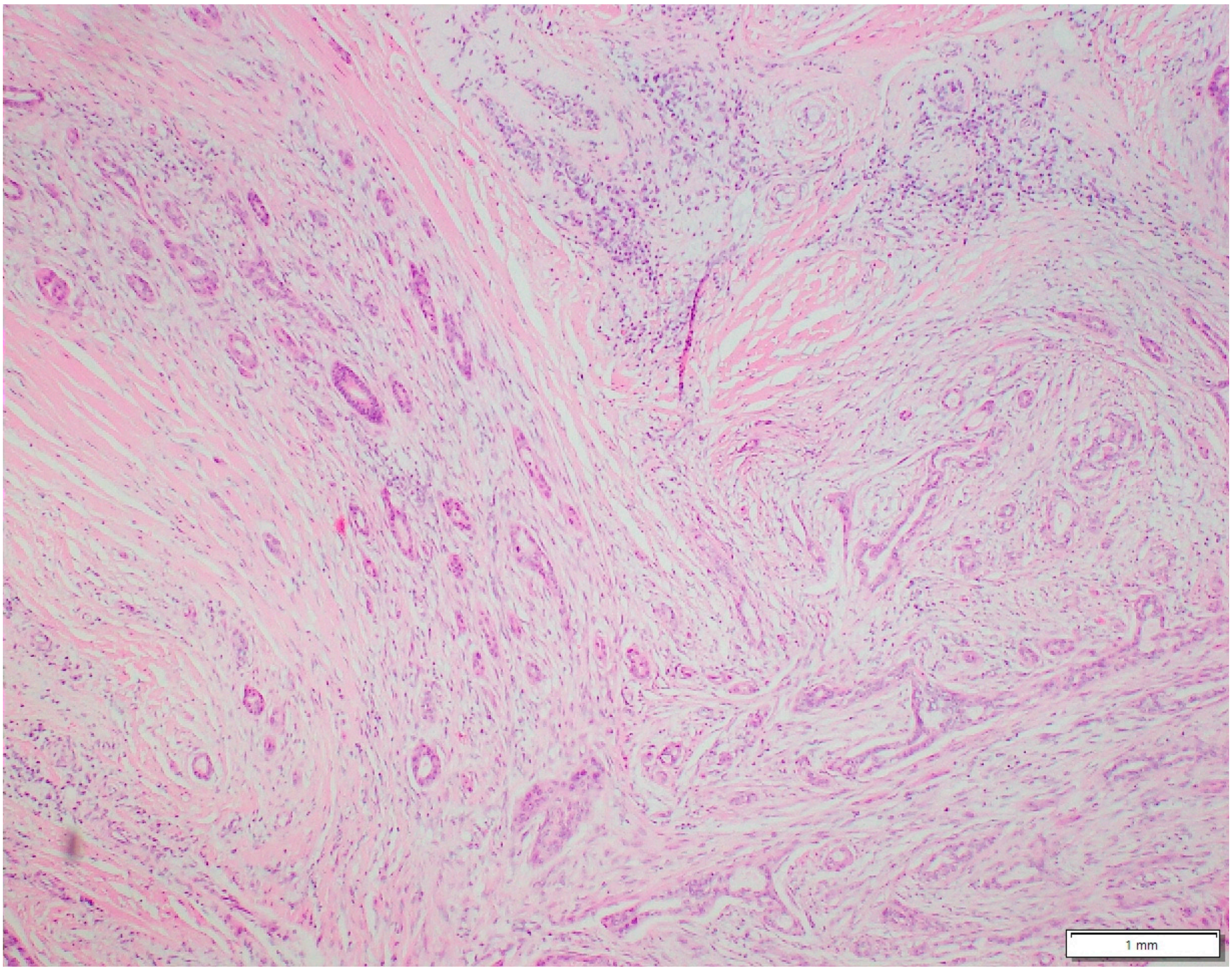

Image S1. Low-grade adenosquamous carcinoma containing infiltrative glands with mild cytology, focal squamoid differentiation, lymphoid aggregates and behind desmoplastic stroma.

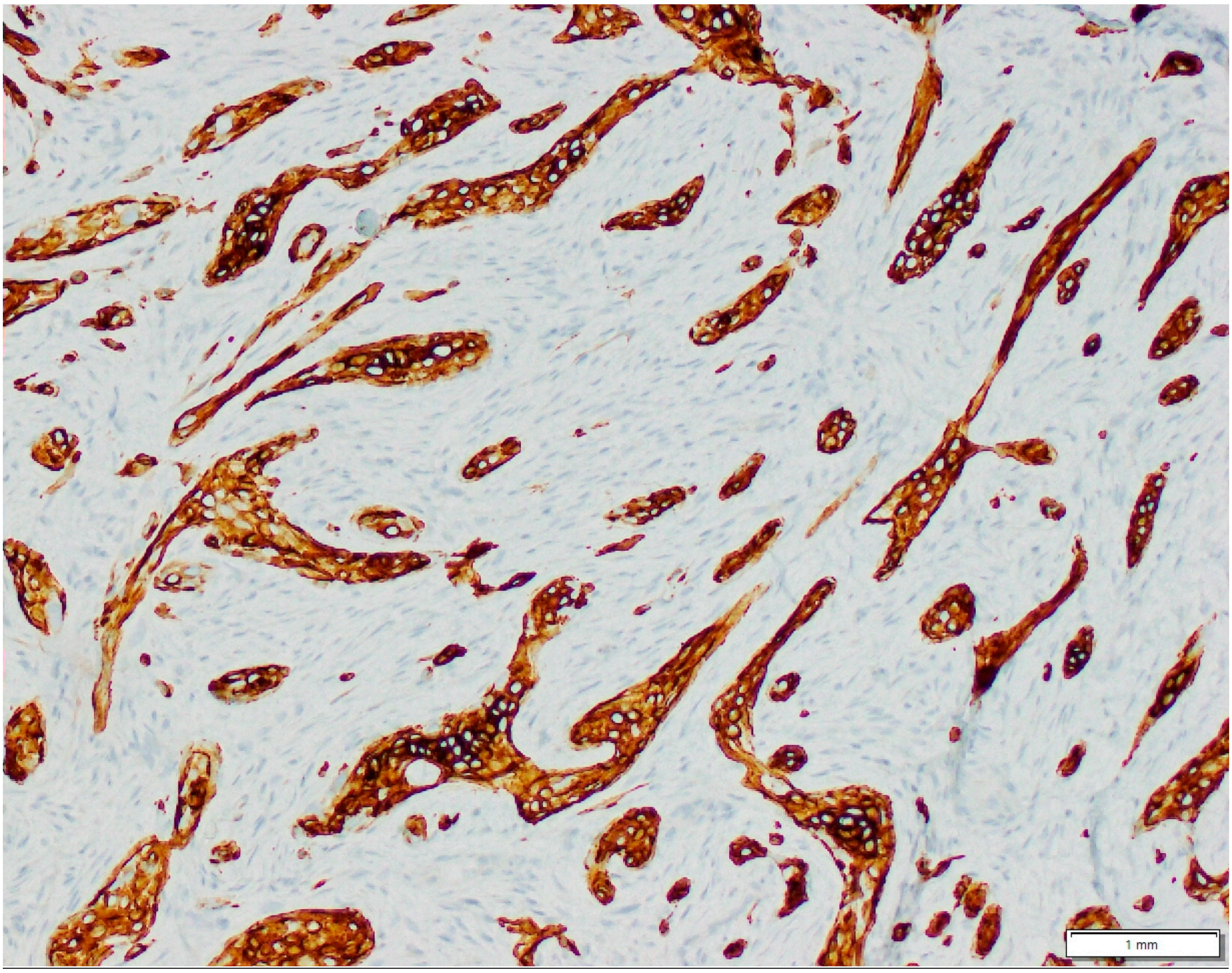

Image S2. Low-grade adenosquamous carcinoma with squamoud differentiation,which stains immunohistorically positive with cytokeratin 7.

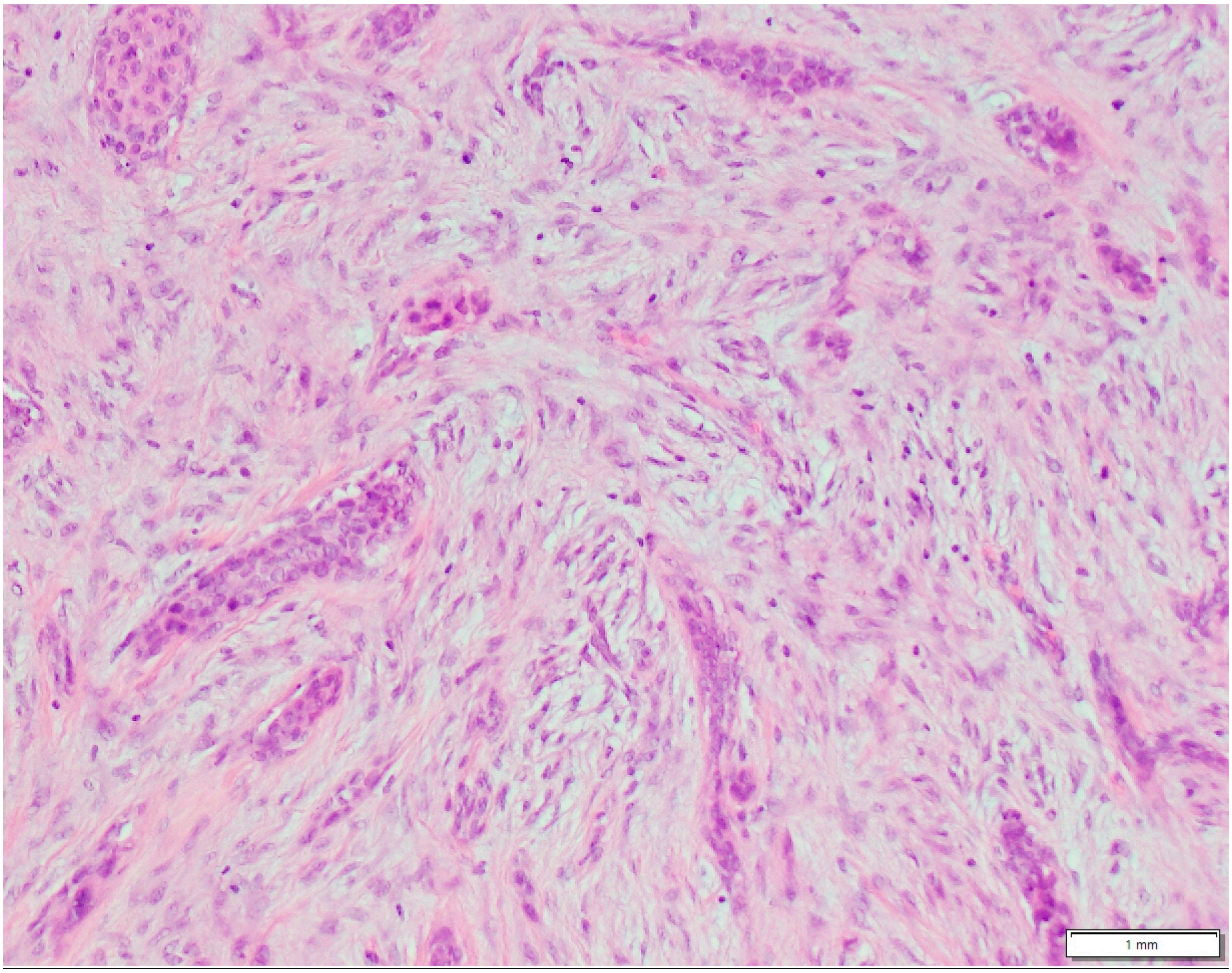

Image S3: Histopathological sample from patient 2.

Patient 3

The sample reveals a tumor forming ducts, with nuclei appearing regular. Mitoses are scarcely observed. Dense lymphocytic infiltrates are seen interspersed within the tumor cells. Cytokeratin 5/6 stains positively in the tumor cells, but p63 remains negative. Estrogen and progesterone receptors are negative in the tumor cells. The proliferation index Ki-67 is 13%. Her2/neu is 0.

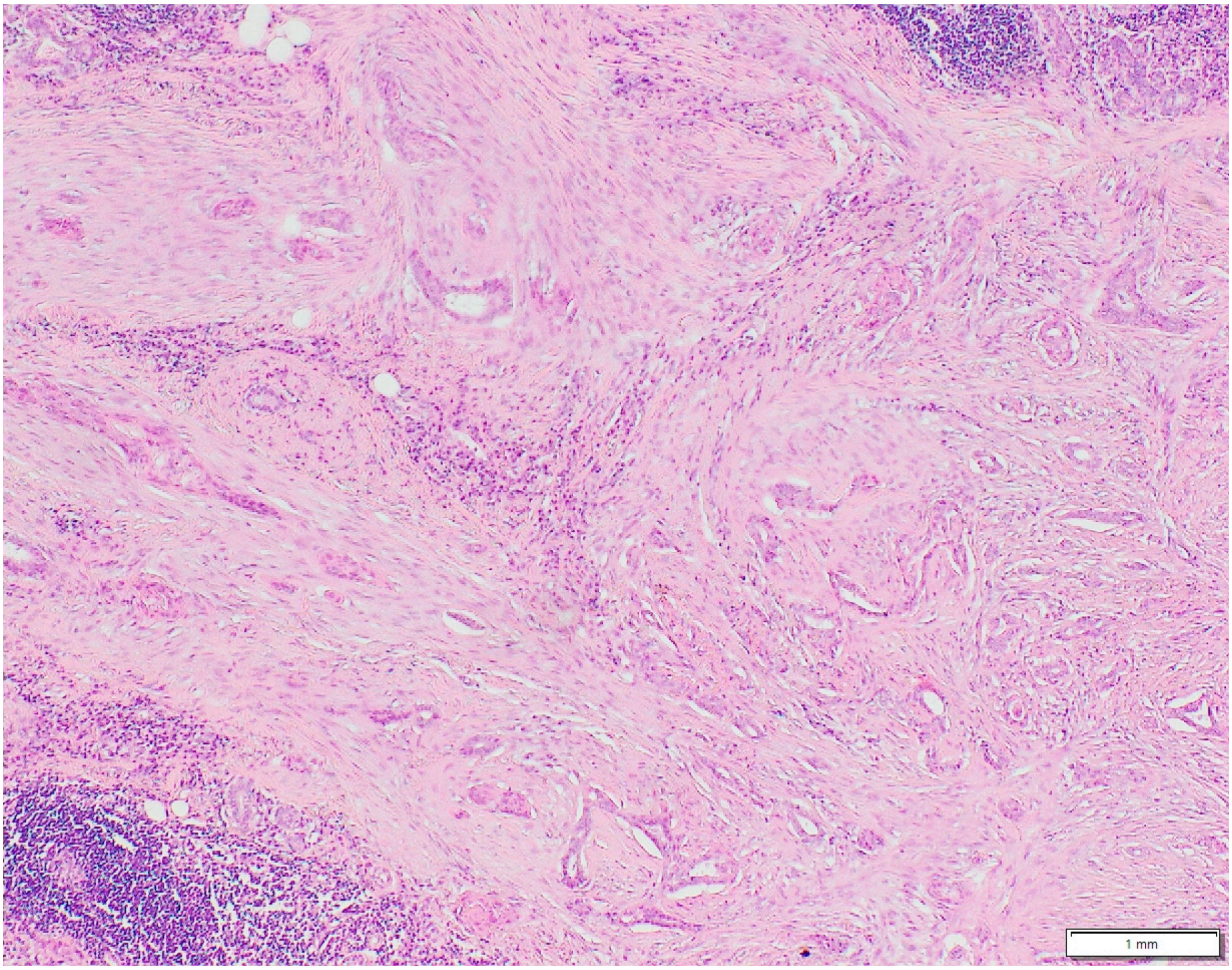

Image S4. Histopathological sample from patient 3.

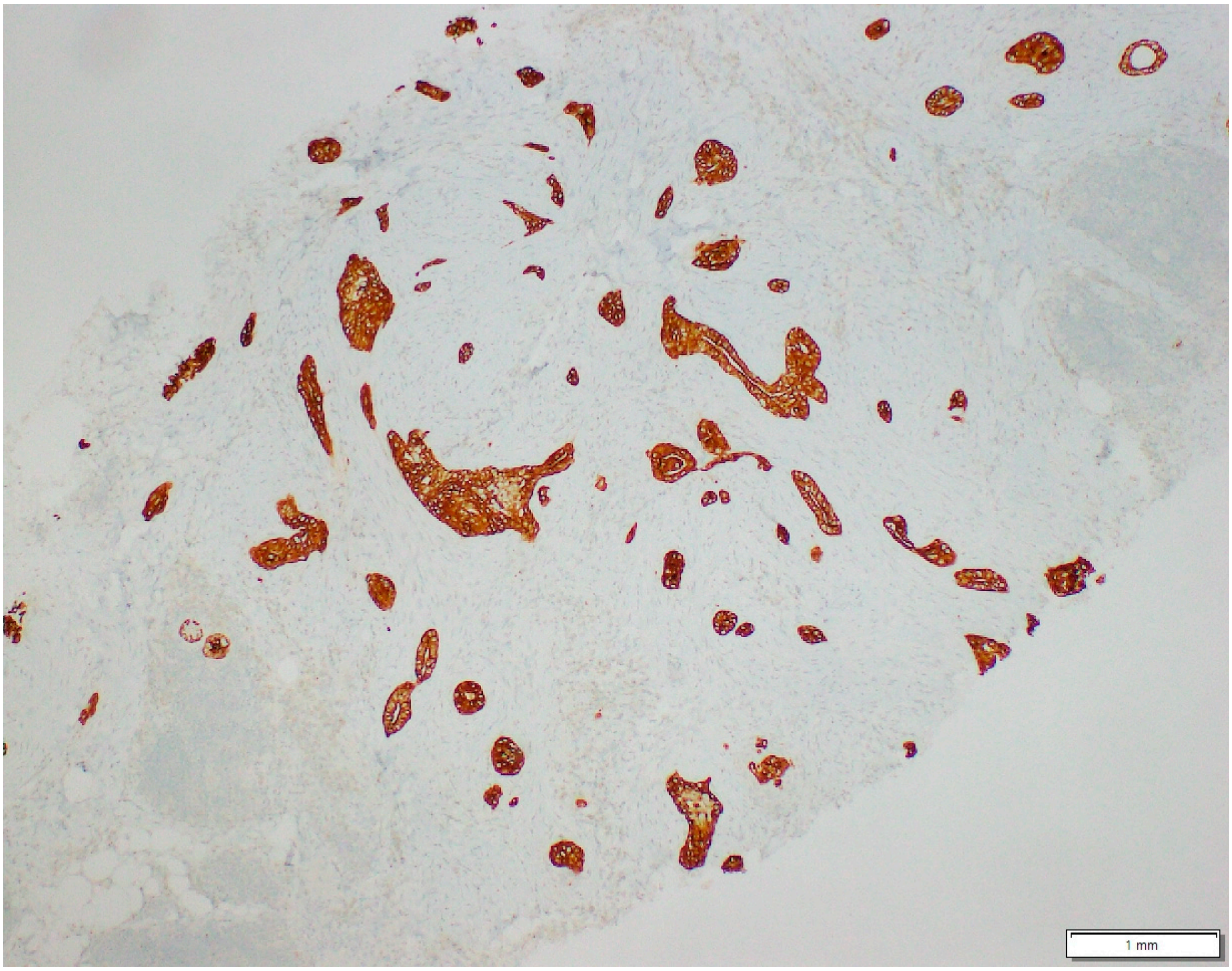

Image S5. The staining shows strong cytokeratin 5/6 positivity.
