# Supplementary material for: Low-Grade Adenosquamous Carcinoma of the Breast: A Single-Center Retrospective Study and a Systematic Literature Review
Source: Cancers (Basel). 2024 Dec 20;16(24):4246. doi: 10.3390/cancers16244246 (PMC11674631; doi:10.3390/cancers16244246)
Supplement: Supplementary file 1 [file cancers-16-04246-s001.zip › Supplementary File S2.pdf]

Study	Study period	Pts.	Age (mean-range)	Presentation	Tumor size (mean, range, mm)	LN metastasis	DM	Breast surgery	Axillary Surgery	RT	CT	HT	Follow-up (months)	Outcome
Rosen et al. (1987)		11	59 (42-76)	11 Mass	23 (15–34) (8 pts)	0/11	0/11	8 Ex, 3 Mt	5 ALND, 6 None	No data	No data	No data	42 (12–72) (for 11 pts.)	4/11 LR 0/11 DM
Van Hoeven et al (1993)		32	61 (33-88)	32 Mass	28 (6 – 86)	1/12	1/32	19 Ex, 13 Mt	12 ALND, 20 None	No data	No data	No data	68 (12-124) (for 25 pts.)	5/25 LR 0/25 LNR 1/25 DM 1/25 DfD
Drudis et al (1994)		19	No data	No data	No data	No data	No data	No data	No data	No data	No data	No data	No data	No data
Krigman et al (1996)		1	57	1 Mass	10	No data	No data	No data	0	0/1	0/1	0/1	No data	No data
Shizawa et al (1997)		1	47	1 Mass	16	0/1	0/1	1 Mt	1 ALND	0/1	0/1	0/1	16	0/1 LR 0/1 LNR 0/1 DM
Ferrara et al (1999)		1	57	1 Mass	14	0/1	0/1	1 Ex	ALND	1/1	0/1	0/1	36	0/1 LR 0/1 LNR 0/1 DM
Denley et al (2000)	1996-1997	4	60 (49-68)	2 Mass 2 Mammogram	14 (7-20)	0/1	No data	1 Ex	No data	No data	No data	No data	No data	No data
Gobbi et al (2003)	1985-2001	6	44 (29-75) (for 5 pt)	5 Mass 1 Mammogram	14 (10-21)	0/1	No data	5 Ex, 1 Mt	1 ALND	No data	No data	No data	42 (1 pt)	0/1 LR 0/1 LNR 0/1 DM
Ho et al (2006)	1996-2005	4	56 (51-62)	3 Mass 1 Mammogram	22 (8-35)	0/4	0/4	2 Ex, 2 Mt	3 ALND, 1 None	0/4	1/4	0/4	42 (24–96) (for 3 pts.)	0/3 LR 0/3 LNR 0/3 DM 0/3 DfD
Noel et al (2010)		1	49	1 follow-up ultrasound	5	0/1	0/1	1 Ex	No data	No data	No data	No data	No data	No data
Geyer et al (2010)	No data	5	70 (54 -76)	No data	27 (15-37)	0/1	0/1	No data	No data	No data	No data	No data	No data	No data
Agrawal et al (2010)		1	19	1 Mass	50	0/1	0/1	1 Mt	1 None	0/1	0/1	0/1	No data	No data
Sironi et al (2012)		1	74	1 Mammogram	22	0/1	0/1	No data	1 SLNB	No data	No data	No data	No data	No data
Kawaguchi et al (2012)	1998-2011	30	53 (20 -85)	29 Mass	12	0/30	0/30	28 Ex	2 SLNB, 28 None	No data	No data	No data	No data	No data

Chuthapisith et al (2013)		1	55	1 Mass	20	0/1	0/1	1 Ex	1 None	0/1	0/1	0/1	48	1/1 LR*****<br>0/1 LNR<br>0/1 DM<br>0/1 DfD
Scali et al (2013)	1989-2010	10	65<br>(30 -81)	5 Mass<br>2 Nipple Symptom<br>3 Mammogram	18<br>(11-28) (7 pts)	0/10	0/10	7 Ex,<br>3 Mt	7 ALND,<br>3 none	7/10	1/10	0/10	78<br>(24 – 138)	0/10 LR<br>0/10 LNR<br>0/10 DM<br>0/10 DfD
Cha et al (2014)		1	69	1 Mass	14	No data	No data	No data	No data	No data	No data	No data	No data	No data
Wilsher et al (2014)		1	45	1 Mass	9	0/1	0/1	1 Mt	No data	No data	No data	No data	No data	No data
Senger et al (2015)		1***	68	1 Mass	No data	0/2	0/1	2 Mt	2 SLNB	0/1	0/1	0/1	48	0/2 LR<br>0/2 LNR<br>0/2 DM<br>0/1 DfD
Bataillon et al (2014) *******														
Tan et al (2015)	2006-2014	8	39<br>(28 -49)	8 Mass	7<br>(0.5-25)	0/8	0/8	5 Ex,<br>3 Mt	5 SLNB,<br>3 none	0/8	1/8	0/8	41<br>(28 - 56)	0/8 LR<br>0/8 LNR<br>0/8 DM<br>0/8 DfD
Wu et al (2017)		1	42	1 Mass	15	0/1	0/1	1 Ex	1 ALND	No data	No data	No data	6	0/1 LR<br>0/1 LNR<br>0/1 DM<br>0/1 DfD
Bataillon et al (2018)	1999-2013	13	53<br>(28 -85)	9 Mass,<br>4 Mammogram	22<br>(3.5-70)	0/13	0/13	12 Ex,<br>1 Mt	6 ALND,<br>3 SLNB	10/13	5/13	0/13	91<br>(36 - 204)	0/13 LR<br>0/13 LNR<br>0/13 DM<br>0/13 DfD
Delgoado Hardegree et al (2018)		1	55	1 Mammogram (palpable)	17	No data	No data	1 Ex	No data	1/1	0/1	0/1	No data	No data
Thornley et al (2020)		1	24	1 Mass	13	0/1	0/1	1 Ex	No data	No data	No data	No data	No data	No data
Wilsher et al (2020)		1	46	1 Nipple symptom	10	No data	No data	1 Ex	No data	No data	No data	No data	No data	No data
Kashu et al (2020)		1	68	1 Mass	8	0/1	0/1	1 Ex	1 none	0/1	0/1	0/1	No data	No data
Nam et al (2020)		1	“in her 40s”	1 Mass, pain	8	0/1	0/1	1 Ex	1 none	1/1	0/1	1/1	No data	No data
Priyadarshini et		1	72	1 Mammogram	10	0/1	0/1	1 Ex	1 none	0/1	0/1	0/1	6	0/1 LR

al (2022)														0/1 LNR<br>0/1 DM<br>0/1 DfD
Sae-Kho et al (2022)	2000-2019	6**	68 (47-84)	6 Mass, 1 Nipple Symptom	No data	0/5	0/5	3 Ex, 4 Mt	1 none	**	**	**	No data	1/5 LR<br>0/5 LNR<br>0/5 DM<br>0/5 DfD
Baldwa et al (2022)		1	85	1 Mass	No data	No data	No data	1 Ex	No data	No data	No data	No data	No data	0/1 LR<br>0/1 LNR<br>0/1 DM<br>0/1 DfD
Cartagena et al (2023) *	2002-2021	34	60 (29-80)	10 Mass, 13 Mammogram, 2 Nipple Symptom	10 (1.4-23)	1 ITC**** + 0/33	0/34	14 Ex, 2 Mt	21 SLNB	12/16 (12/14 after Ex)	0/16	8/16	43 (2-115) (for 16 pts.)	1/16 LR<br>0/16 LNR<br>0/16 DM<br>0/16 DfD
Abrari et al (2023)		1	55	1 Mass	38	0/1	0/1	1 Ex	1 SLNB	0/1	0/1	0/1	15	0/1 LR<br>0/1 LNR<br>0/1 DM<br>0/1 DfD
Lewis et al (2023)	1990-2021	25	66 (35-85)	18 Mass, 3 Mammogram, 1 Coindidental Finding, 3 No data	15 (4-46)	0/25	0/25	18 Ex/4 Mt, 3 biopsy	11 SLNB	5/25	0/25	0/25	24 (1-108)	0/25 LR<br>1/25 LNR******<br>0/25 DM<br>0/25 DfD
Laokulrath et al (2024)		1	53	1 Mass	23	0/1	0/1	1 Ex	1 none	0/1	0/1	0/1	4	0/1 LR<br>0/1 LNR<br>0/1 DM<br>0/1 DfD

Supplementary Table S1. Summary of the studies reporting data of patients with low-grade adenosquamous carcinoma. Ex = excision/breast conserving surgery, Mt = mastectomy, ALND = axillary lymph node dissection, SLNB = sentinel lymph node biopsy.

*conference abstract

** the series included a seventh patient with high-grade ASC with distant metastasis at the time of diagnosis, but the data of this patient is not reported separately. The data is given partly for 7 patients but only 6 cases are reported.

*** bilateral LGASC

**** patient with ITC presented with concurrent ipsilateral DCIS

*****recurrence as intermediate-grade ASC

******One patient who refused surgery and adjuvant treatment presented with lymph node metastasis 18 months after the diagnosis. The patient refused treatment for metastasis. The patient is reported to be alive 19 months after the metastasis was detected.

*******The data is included in Bataillon et al (2018)

REFERENCES

1. Rosen PP, Ernsberger D. Low-grade adenosquamous carcinoma. A variant of metaplastic mammary carcinoma. Am J Surg Pathol. 1987;11(5):351–8. Available from: https://pubmed.ncbi.nlm.nih.gov/3578645/
2. Van Hoeven KH, Drudis T, Cranor ML, Erlandson RA. Low-Grade Adenosquamous Carcinoma of the Breast. A Clinocopathologic Study of 32 Cases with Ultrastructural Analysis. Am J Surg Pathol. 1993;17(3):248–58. Available from: https://journals.lww.com/ajsp/pages/articleviewer.aspx?year=1993&issue=03000&article=00005&type=Abstract
3. Drudis T, Arroyo C, Van Hoeven K, Cordon-Cardo C, Rosen PP. The pathology of low-grade adenosquamous carcinoma of the breast. An immunohistochemical study. Pathol Annu. 1994;29 (Pt 2):181-97. PMID: 7936747.
4. Krigman HR, Iglehart JD, Coogan AC, Layfield LJ. Fine-needle aspiration of low grade adenosquamous carcinoma of the breast. Diagn Cytopathol. 1996 Jun;14(4):321-4. doi: 10.1002/(SICI)1097-0339(199605)14:4<321::AID-DC8>3.0.CO;2-I. PMID: 8725132.
5. Shizawa S, Sasano H, Suzuki T, Ishii H, Takeda T, Nagura H. Low-grade adenosquamous carcinoma of the breast: A case report with cytologic findings and review of the literature. Pathol Int. 1997 Apr 1 ;47(4):264–7. Available from: https://onlinelibrary.wiley.com/doi/full/10.1111/j.1440-1827.1997.tb04490.x
6. Ferrara G, Nappi O, Wick MR. Fine-needle aspiration cytology and immunohistology of low-grade adenosquamous carcinoma of the breast. Diagn Cytopathol. 1999 Jan;20(1):13-8. doi: 10.1002/(sici)1097-0339(199901)20:1<13::aid-dc4>3.0.co;2-#. PMID: 9884821.
7. Denley H, Pinder SE, Tan PH, Sim CS, Brown R, Barker T, Gearty J, Elston CW, Ellis IO. Metaplastic carcinoma of the breast arising within complex sclerosing lesion: a report of five cases. Histopathology. 2000;36(3):203–9. Available from: https://pubmed.ncbi.nlm.nih.gov/10692021/
8. Gobbi H, Simpson JF, Jensen RA, Olson SJ, Page DL. Metaplastic spindle cell breast tumors arising within papillomas, complex sclerosing lesions, and nipple adenomas Mod Pathol. 2003. 16:893-901. doi: 10.1097/01.MP.0000085027.75201.B5
9. Ho BC, Tan HW, Lee VK, Tan PH. Preoperative and intraoperative diagnosis of low-grade adenosquamous carcinoma of the breast: potential diagnostic pitfalls Histopathology. 2006. 49:603-611. doi: 10.1111/j.1365-2559.2006.02524.x

10. Noel JC, Buxant F, Engohan-Aloghe C. Low-grade adenosquamous carcinoma of the breast--A case report with a BRCA1 germline mutation Pathol Res Pract. 2010. 206:511-513. doi: 10.1016/j.prp.2010.01.008
11. Geyer FC, Lambros MB, Natrajan R, Mehta R, Mackay A, Savage K, Parry S, Ashworth A, Badve S, Reis-Filho JS. Genomic and immunohistochemical analysis of adenosquamous carcinoma of the breast Mod Pathol. 2010. 23:951-960. doi: 10.1038/modpathol.2010.82
12. Agrawal A, Saha S, Ellis IO, Bello AM. Adenosquamous carcinoma of breast in a 19 years old woman: a case report. World J Surg Oncol. 2010 May 27;8(1):1–3. Available from: https://wjso.biomedcentral.com/articles/10.1186/1477-7819-8-44
13. Sironi M, Lanata S, Pollone M, Saro F. Fine-needle aspiration cytology of low-grade adenosquamous carcinoma of the breast. Diagn Cytopathol. 2012 Aug 1;40(8):713–5. Available from: https://onlinelibrary.wiley.com/doi/full/10.1002/dc.21709
14. Kawaguchi K, Shin SJ. Immunohistochemical staining characteristics of low-grade adenosquamous carcinoma of the breast. Am J Surg Pathol. 2012 Jul;36(7):1009–20. Available from: https://journals.lww.com/ajsp/fulltext/2012/07000/immunohistochemical_staining_characteristics_of.10.aspx
15. Chuthapisith S, Warnnissorn M, Amornpinyokiat N, Pradniwat K, Angsusinha T. Metaplastic carcinoma of the breast with transformation from adenosquamous carcinoma to osteosarcomatoid and spindle cell morphology. Oncol Lett. 2013 Sep 1;6(3):728–32. Available from: http://www.spandidos-publications.com/10.3892/ol.2013.1464/abstract
16. Scali EP, Ali RH, Hayes M, Tyldesley S, Hassell P. Low-grade adenosquamous carcinoma of the breast: Imaging and histopathologic characteristics of this rare disease. Can Assoc Radiol J. 2013 Nov 1;64(4):339–44. Available from: https://journals.sagepub.com/doi/full/10.1016/j.carj.2012.09.002?casa_token=x2VerupY9DgAAAAA%3AcHsl_4O5pxV_j4FbcrfA-dc9WixbOZgu0zik2jCMlDxfupaLY22OdpN7QflHPtHiZ5nd059RMX3i6DM
17. Cha YJ, Kim GJ, Park BW, Koo JS. Low-grade adenosquamous carcinoma of the breast with diverse expression patterns of myoepithelial cell markers on immunohistochemistry: a case study Korean J Patho. 2014. 48:229-233. doi: 10.4132/KoreanJPathol.2014.48.3.229
18. Wilsher MJ, Desai AJ, Pinder SE. Low-grade adenosquamous carcinoma arising in association with a nipple adenoma Histopathology. 2020. 76:784-787. doi: 10.1111/his.14033
19. Senger JL, Meiers P, Kanthan R. Bilateral synchronous low-grade adenosquamous carcinoma of the breast: A Case report with review of the current literature. Int J Surg Case Rep. 2015 Jul 27;14:53–7. Available from: https://pubmed.ncbi.nlm.nih.gov/26218176/
20. Bataillon G, Collet J-F, Voillemot N, Menet E, Vincent-Salomon A, Klijanienko J; Fine-Needle Aspiration of Low-Grade Adenosquamous Carcinomas of the Breast: A Report of Three New Cases. Acta Cytologica 1 October 2014; 58 (5): 427–431. https://doi.org/10.1159/000367585

21. Tan QT, Chuwa EW, Chew SH, Lim-Tan SK, Lim SH. Low-grade adenosquamous carcinoma of the breast: A diagnostic and clinical challenge Int J Surg. 2015. 19:22-26. doi: 10.1016/j.ijsu.2015.05.010
22. Wu HB, Zhang AL, Wang W, Li H. Expression of hormone receptors in low-grade adenosquamous carcinoma of the breast: A case report Medicine. 2017. Nov;96(46):e8785. doi: 10.1097/MD.0000000000008785
23. Bataillon G, Fuhrmann L, Girard E, Menet E, Laé M, Capovilla M, Treilleux I, Arnould L, Penault-Llorca F, Rouzier R, et al. High rate of PIK3CA mutations but no TP53 mutations in low-grade adenosquamous carcinoma of the breast. Histopathology. 2018 Aug 1;73(2):273–83. Available from: https://pubmed.ncbi.nlm.nih.gov/29537649/
24. Delgado Hardegree LA, Ferguson PE, Gulla S. Low-grade Adenosquamous Carcinoma of the Breast: A Case Report. Semin Roentgenol. 2018 Oct 1;53(4):249–51.
25. Thornley L, Nayler S, Benn CA. Low-grade adenosquamous carcinoma of the breast. Breast J. 2020 Sep 1;26(9):1845–6. Available from: https://pubmed.ncbi.nlm.nih.gov/32279426/
26. Wilsher MJ. Adenosquamous proliferation of the breast and low grade adenosquamous carcinoma: a common precursor of an uncommon cancer? Pathology. 2014 Aug 1;46(5):402–10.
27. Kashu N, Oura S, Yoshida H, Nishino E, Makimoto S. A Case of Squamous Cell Carcinoma of the Breast with Low-Grade Adenosquamous Carcinoma. Case Rep Oncol. 2020 Sep 23;13(3):1152–7.
28. Nam G, Strenger R, Cutitar M, Wang Y. Low-grade adenosquamous carcinoma of the breast: A case with pathogenic germline mutation in the BRIP1 gene. Hum Pathol Case Reports. 2020 Nov 1;22:200444.
29. Priyadarshini S, Singh N, Ali H, Kasireddy V. Adenosquamous Carcinoma of the Breast: Case Report and Literature Review. Cureus. 2022 Jun 14;14(6):e25940. doi: 10.7759/cureus.25940. PMID: 35711248; PMCID: PMC9197327
30. Sae-Kho TM, Bhatt A, Solanki MH, Jeans EB, Corbin KS, Fazzio RT, Glazebrook KN. Imaging features of adenosquamous carcinoma of the breast – A rare variant of metaplastic breast carcinoma. BJR | Case Reports. 2022 Nov;7(6):20210108. Available from: https://pmc.ncbi.nlm.nih.gov/articles/PMC8906157/
31. Baldwa V, Nizamovski J. Low-grade adenosquamous carcinoma of breast – case report. Int J Sci Res. 2022 Jul 1;10–1.
32. Cartagena LC, Brogi E, Reis-Filho J, Wen H. Low grade adenosquamous carcinoma of the breast: A clinicopathologic review of 34 cases. Mod Pathol. 2022 Mar 21;35(Suppl. 2).
33. Abrari A, Shehwar D, Akhtar K. Low-grade adenosquamous carcinoma of the breast: A rare case presentation. J Cancer Res Ther. 2023 Jan 1;19(2):480–3.
34. Lewis G, Fong N, Gjeorgjievski SG, Li XB, Li Z, Wei S, Sturgis CD, Wang C, Komforti M, Zhang H, et al. Low-grade adenosquamous carcinoma of the breast: a clinical, morphological and immunohistochemical analysis of 25 patients. Histopathology. 2023 Aug 1;83(2):252–63. Available from: https://onlinelibrary.wiley.com/doi/full/10.1111/his.14917

35. Laokulrath N, Chuwa E, Gudi M, Tan PH. Low-Grade Adenosquamous Carcinoma of the Breast Masquerading as a Fibroepithelial Lesion on Core Biopsy: A Challenging Case. Pathobiology. 2024 Jul 17;1–8. Available from: https://pubmed.ncbi.nlm.nih.gov/38952139/
